# Histone deacetylase inhibitor potentiated the ability of MTOR inhibitor to induce autophagic cell death in Burkitt leukemia/lymphoma

**DOI:** 10.1186/1756-8722-6-53

**Published:** 2013-07-18

**Authors:** Li Hua Dong, Shu Cheng, Zhong Zheng, Li Wang, Yang Shen, Zhi Xiang Shen, Sai Juan Chen, Wei Li Zhao

**Affiliations:** 1 State Key Laboratory of Medical Genomics, Shanghai Institute of Hematology, Shanghai Rui Jin Hospital, Shanghai Jiao Tong University School of Medicine, Shanghai, China; 2 Pôle de Recherches Sino-Français en Science du Vivant et Génomique, Laboratory of Molecular Pathology, Shanghai, China

**Keywords:** Histone deacetylase inhibitor, MTOR inhibitor, Autophagy, Burkitt leukemia/lymphoma, MYC

## Abstract

**Background:**

Burkitt leukemia/lymphoma is a major subtype of aggressive B-cell lymphoma. Biological targeted therapies on this disease need to be further investigated and may help to improve the clinical outcome of the patients.

**Methods:**

This study examined the anti-tumor activity of the histone deacetylases (HDAC) inhibitor valproic acid (VPA) combined with the mammalian target of rapamycin (MTOR) inhibitor temsirolimus in Burkitt leukemia/lymphoma cell lines, as well as in primary tumor cells and a murine xenograft model.

**Results:**

Co-treatment of VPA and temsirolimus synergistically inhibited the tumor cell growth and triggered the autophagic cell death, with a significant inhibition of MTOR signaling and MYC oncoprotein. Functioned as a class I HDAC inhibitor, VPA potentiated the effect of temsirolimus on autophagy through inhibiting HDAC1. Molecular silencing of HDAC1 using small interfering RNA (siRNA) attenuated VPA-mediated regulation of CDKN1A, CDKN1B and LC3-I/II, regression of tumor cell growth and induction of autophagy. Meanwhile, VPA counteracted temsirolimus-induced AKT activation via HDAC3 inhibition. HDAC3 siRNA abrogated the ability of VPA to modulate AKT phosphorylation, to suppress tumor cell growth and to induce autophagy. Strong antitumor effect was also observed on primary tumor cells while sparing normal hematopoiesis ex vivo. In a murine xenograft model established with subcutaneous injection of Namalwa cells, dual treatment efficiently blocked tumor growth, inhibited MYC and induced in situ autophagy.

**Conclusions:**

These findings confirmed the synergistic effect of the HDAC and MTOR inhibitors on Burkitt leukemia/lymphoma, and provided an insight into clinical application of targeting autophagy in treating MYC-associated lymphoid malignancies.

## Background

Burkitt leukemia/lymphoma (BL) is a highly aggressive subtype of B-cell neoplasm characterized by constitutive MYC expression and PI3K activation [1]. Although BL responds to intensive chemotherapy regimens, biologically targeted therapies should be developed, especially in high-risk patients and in the setting of relapsed/refractory disease [2].

Autophagy is generally involved in cancer progression [3]. Although autophagy is primarily a cell protective process, it can also induce cell death, known as programmed cell death type II [4]. Experimental study showed that mice with heterozygous disruption of *BECN1* present decreased autophagy and are more prone to the development of spontaneous tumors including lymphomas [5]. Clinically, defect in autophagy is also related to aggressive phenotype and poor prognosis in lymphoma patients [6,7]. These results indicated that reactivation of autophagy could be mechanistically important in lymphoma treatment.

Signal transduction inhibitors become an emerging therapeutic option for molecular tumor targeting [8]. Mammalian target of rapamycin (MTOR) signaling plays a major role in tumor cell growth and is aberrantly activated in lymphoma [9,10]. MTOR inhibitors possess single-agent therapeutic activity, but drug resistance is frequently observed [10]. Thus, unique combination to enhance the effect of MTOR inhibitors is particularly attractive [11].

Histone deacetylase (HDAC) inhibitors constitute a group of compounds that promote histone acetylation, chromatin uncoiling and downmodulation of genes involved in cancer [12]. Widely used as an anti-convulsant, valproic acid (VPA) belongs to the short chain fatty acid HDAC inhibitors and possesses anti-tumor activity [13]. It negatively regulates B-lymphoma cell proliferation and shows therapeutic potential on refractory patients at the standard dose [14,15]. Although simultaneous inhibition of MTOR and HDAC exerts profound anti-tumor properties, the possible interaction and therapeutic mechanism of this combination remain to be defined in BL.

To address this issue, we examined the combinatorial action of the HDAC inhibitor VPA with clinical relevant MTOR inhibitor temsirolimus in BL cells both in vitro and in vivo. These two agents interacted in a synergistic manner to induce autophagic cell death in BL cells, in association with a significant inhibition of MTOR pathway and MYC oncoprotein.

## Results

### Combination of the HDAC inhibitor VPA with the MTOR inhibitor temsirolimus induced synergistic cytotoxicity in BL cells

**Figure 1:**
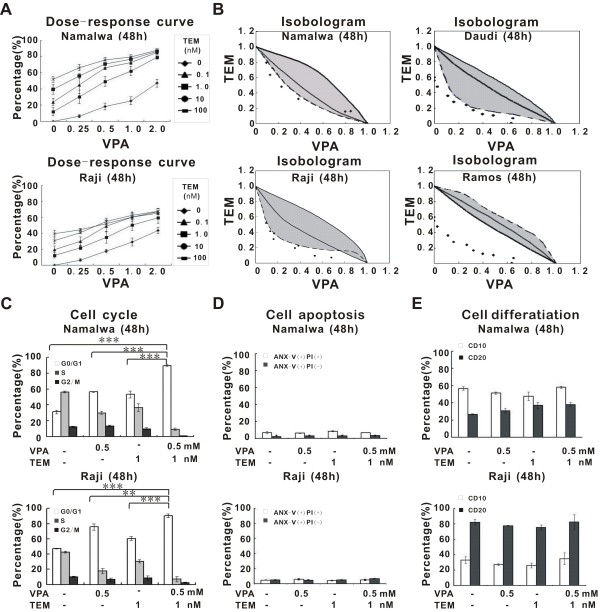
**Valproic acid combined with temsirolimus inhibited Burkitt leukemia/lymphoma (BL) cell growth. (A)** In BL cell line Namalwa and Raji, co-treatment of valproic acid (VPA) with temsirolimus (TEM) induced significant growth inhibition, comparing with each agent alone. **(B)** Isobolographic curve showed that the data points lie to the left of the envelope of additivity (gray area), indicating that the combination is synergistic in BL cell lines Namalwa, Raji, Daudi and Ramos. **(C-E)** When VPA (0.5mM) was combined with TEM (1 nM), the percentages of G0/G1 phase cells were significantly increased **(C)**, without obvious change of cell apoptosis **(D)** or differentiation **(E)**. ***P<0.001 and **P<0.01 comparing with the combination group.

The BL cell lines Namalwa and Raji were treated with different concentrations of VPA and/or temsirolimus for 48 hours. Dose–response curves were shown in Figure 1A. Compared with each agent alone, a marked increase in cell growth inhibition was observed with combined treatment. For example, 0.5 mM VPA and 1 nM temsirolimus alone induced approximately 20% reduction in cell viability. However, in combination they achieved more than a 60% cell reduction. Isobolographic analysis yielded most of the data points to the left of the envelope of additivity, denoting highly synergistic interactions in both cell lines. Similar results were obtained with other BL cell lines (Daudi and Ramos, Figure 1B). The synergistic effect was further confirmed by the Calcusyn software (Additional file 1: Figure S1).

In Namalwa and Raji cells, cell cycle analysis revealed significantly higher percentage of G0/G1-phase cells in the combination group than in the single-agent and the control group, indicating that co-treatment of VPA (0.5 mM) and temsirolimus (1 nM) synergistically inhibited BL cell growth through cell cycle arrest (Figure 1C). Cell apoptosis and differentiation-related antigens were also evaluated. There was no obvious change in the percentage of ANX-V-positive cells (Figure 1D), CD10- or CD20-expressing cells (Figure 1E) treated with VPA (0.5 mM) and/or temsirolimus (1 nM) for 48 hours. The apoptosis-related c-caspase-3 and c-PARP expression were not detected during treatment (Additional file 2: Figure S2).

### VPA combined with temsirolimus synergistically triggered autophagic cell death and inhibited MTOR pathway

**Figure 2:**
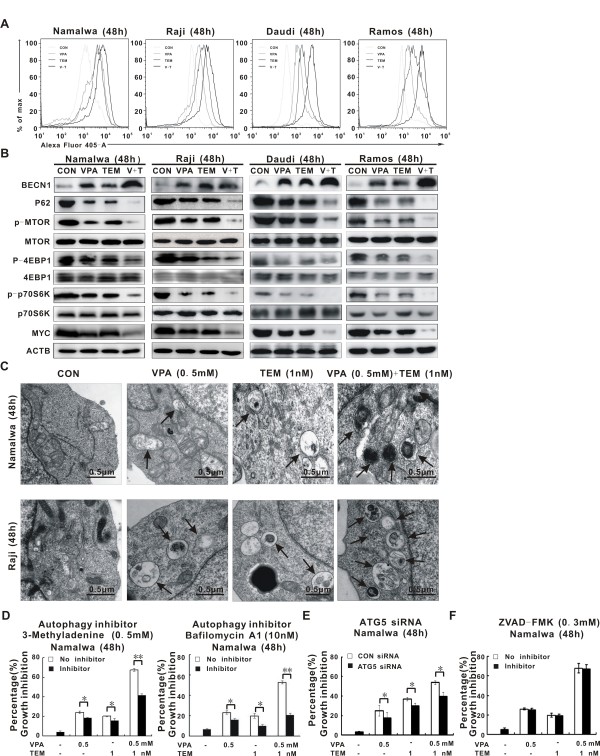
**Valproic acid combined with temsirolimus induced Burkitt leukemia/lymphoma (BL) cell autophagy. (A)** As detected by flow cytometry, co-treatment of valproic acid (VPA, 0.5 mM) with temsirolimus (TEM, 1nM) increased LC3 intensity in BL cell lines Namalwa, Raji, Daudi and Ramos. **(B)** Western blot analysis revealed that combined treatment increased BECN1 expression, but decreased expression of P62, p-MTOR, p-4EBP1, p-P70S6K and MYC. **(C)** Ultrastructural study of Namalwa and Raji cells showed that combined treatment induces more frequently BL cell autophagy than each agent alone. **(D-F)** Growth inhibition of Namalwa cells was significantly reduced by autophagy inhibitor 3-Methyladenine (0.5 mM) and Bafilomycin A1 (10 nM) **(D)**, **P<0.01, *P<0.05 comparing with no inhibitor), by molecular silencing of the ATG5 siRNA (**E**, *P<0.05 comparing with CON siRNA), but not by pan-caspase inhibitor ZVAD-FMK (0.3 mM) **(F)**.

As shown in Figure 2A, significantly higher LC3 intensity was observed in BL cells co-treated with VPA (0.5 mM) and temsirolimus (1 nM), than those of the control cells and cells treated with VPA or temsirolimus alone. Western blot analysis revealed that VPA in conjunction with temsirolimus increased the expression of autophagosome-associated BECN1, comparing with the single-agent group. Also the autophagic flux was confirmed by a corresponding decrease in P62 expression, which can be degraded by autophagy (Figure 2B) [16].

MTOR is the target of temsirolimus and critically regulates cell autophagy [17]. Whereas VPA and temsirolimus administered individually exert an inhibitory effect, combined treatment inhibited the phosphorylation of MTOR in a more profound manner. Similar patterns were also found in the downstream effectors of MTOR (p-4EBP1 and p-P70S6K), with their total levels remained constantly. Of note, MYC, the oncoprotein of BL, was significantly downregulated in the combination group than in the single-agent group (Figure 2B).

In Namalwa and Raji cells, ultrastructure of tumor cells was studied. Accordingly, typical autophagosomes, present in the single-agent group, were more frequently observed in the combination group (Figure 2C). Synergistic cytotoxicity was significantly diminished by pharmacological autophagy inhibitors (3-Methyladenine and Bafilomycin A1, Figure 2D) and molecular silencing of autophagy (ATG5 siRNA, Figure 2E), but not by pan-caspase inhibitor ZVAD-FMK (Figure 2F), further referring it as autophagy-dependent.

**Figure 3:**
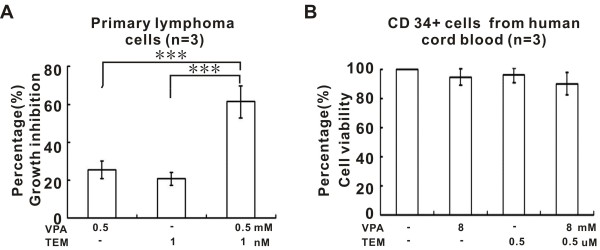
**Valproic acid combined with temsirolimus inhibited primary Burkitt leukemia/lymphoma (BL) cell growth without affecting the proliferation capacity of normal hematopoietic progenitor cells.** Valproic acid (VPA, 0.5 mM) combined with temsirolimus (TEM, 1 nM) inhibited the growth of primary BL cells **(A)**, whereas the proliferation of normal progenitor cells was not affected by VPA (up to 8 mM) and TEM (up to 0.5 μM) **(B)**.

In addition to BL cell lines, co-treatment with VPA (0.5 mM) and temsirolimus (1 nM) significantly inhibited cell growth and results in >60% cell inhibition in primary BL cells (Figure 3A). However, proliferation of normal hematopoietic precursors isolated from human cord blood was not affected even at the concentrations up to 8 mM VPA combined with 0.5 μM temsirolimus (Figure 3B).

### VPA enhanced the effect of temsirolimus on autophagy through inhibiting HDAC1

**Figure 4:**
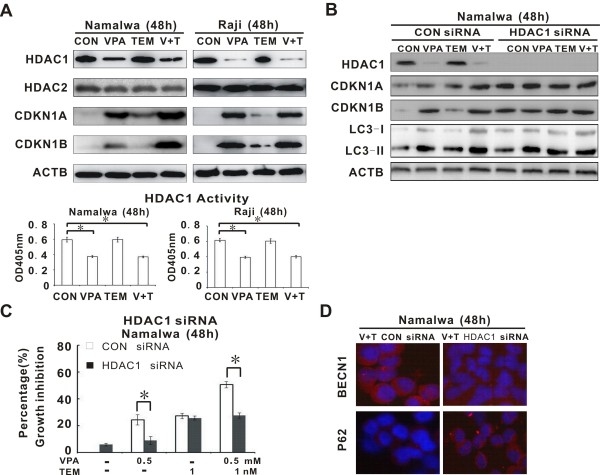
**Valproic acid potentiated temsirolimus to trigger Burkitt leukemia/lymphoma (BL) cell autophagy through inhibiting HDAC1. (A)** Valproic acid (VPA), either alone or in combination, inhibited HDAC1 expression (upper panel), in parallel with decreased HDAC1 enzymatic activity (lower panel), but increased CDKN1A and CDKN1B expression (upper panel). **(B)** Comparing with the negative control (CON siRNA), VPA failed to alter CDKN1A, CDKN1B, or LC3-I/II expression in Namalwa cells transfected with HDAC1 siRNA. **(C** and **D)** HDAC1 siRNA abrogated VPA-induced inhibition of tumor cell growth **(C**, *P<0.05 comparing with CON siRNA) and induction of autophagy **(D**, as detected by immunefluorescence study on BECN1 and P62).

To determine the possible mechanism of synergic effect of VPA and temsirolimus in BL cells, the expression of the main class I HDACs regulated by VPA were assessed by Western blot. The results showed that VPA, either alone or in combination, inhibited HDAC1 but increased CDKN1A and CDKN1B expression, while temsirolimus exerted minimal effects. Decreased HDAC1 expression was associated with reduced enzymatic activity of HDAC1 (Figure 4A). In Namalwa cells transfected with the specific HDAC1 siRNA, CDKN1A, CDKN1B, and LC3-I/II expression were significantly increased and could no longer be altered by VPA treatment, which was found in those transfected with the CON siRNA (Figure 4B). Consequently, HDAC1 siRNA significantly diminished VPA-mediated inhibition of tumor cell growth (Figure 4C) and activation of autophagic cell death (Figure 4D). Collectively, these data implicated that VPA-initiated HDAC1 inhibition was essential for VPA to impart its autophagy-enhancing effect with temsirolimus in BL.

Inhibitory effect of HDAC1 and HDAC2 were obtained in BL cells treated with class I/II HDAC inhibitor suberoylanilide hydroxamic acid (SAHA, Additional file 3: Figure S3).

### VPA counteracted temsirolimus-mediated AKT activation via HDAC3 inhibition

**Figure 5:**
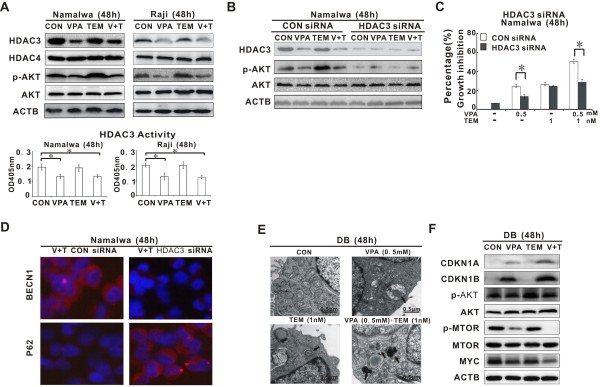
**Valproic acid overcame temsirolimus-mediated AKT activation via HDAC3 inhibition. (A)** Valproic acid (VPA), either alone or in combination, inhibited HDAC3 expression (upper panel), in parallel with decreased HDAC3 enzymatic activity (lower panel) and p-AKT expression (upper panel). **(B)** Compared with the negative control (CON siRNA), VPA failed to alter p-AKT expression in Namalwa cells transfected with HDAC3 siRNA. **(C** and **D)** Tumor cell growth inhibition **(C**, *P<0.05 comparing with CON siRNA) and autophagic cell death **(D**, as detected by immunefluorescence study on BECN1 and P62) were less affected by VPA in Namalwa cells transfected with HDAC3 siRNA than in those transfected with CON siRNA. **(E)** In MYC-expressing diffuse large B-cell lymphoma cell line DB, characteristic changes of autophagy were more frequently detected by ultrastructural study in the combination group than in the single agent group. **(F)** VPA (0.5 mM) combined with temsirolimus (TEM, 1 nM) significantly increased CDKN1A and CDKN1B, decreased p-AKT, p-MTOR and MYC oncoprotein.

As shown in Figure 5A, temsirolimus increased the phosphorylation of AKT, which may lead to feedback activation of MTOR. In VPA-treated cells, HDAC3 expression was downregulated, in parallel with reduced enzymatic activity of HDAC3 and decreased level of p-AKT, while the total AKT remained constantly (Figure 5A).

To validate the role of HDAC3 in AKT dephosphorylation/inactivation, Namalwa cells were transfected with HDAC3 siRNA. Comparing with the CON siRNA, specific knock-down of HDAC3 resulted in a significant decrease of AKT phosphorylation, which could no longer be altered by VPA treatment (Figure 5B). Meanwhile, HDAC3-depleted Namalwa cells were relatively resistant to VPA-mediated cell growth inhibition (Figure 5C) and autophagy induction (Figure 5D). These results indicated that VPA could inhibit HDAC3 and prevent AKT activation. Inhibitory effect of HDAC3 and HDAC4 were obtained in BL cells treated with SAHA (Additional file 3: Figure S3).

Interestingly, when co-treated with VPA (0.5 mM) and temsirolimus (1 nM), MYC-expressing diffuse large B-cell lymphoma (DLBCL) cell line DB was also sensitive to autophagy (Figure 5E), in consistent with increased expression of CDKN1A and CDKN1B, as well as decreased expression of p-AKT, p-MTOR and MYC oncoprotein (Figure 5F).

### Co-treatment of VPA and temsirolimus inhibited tumor growth in a murine xenograft model

**Figure 6:**
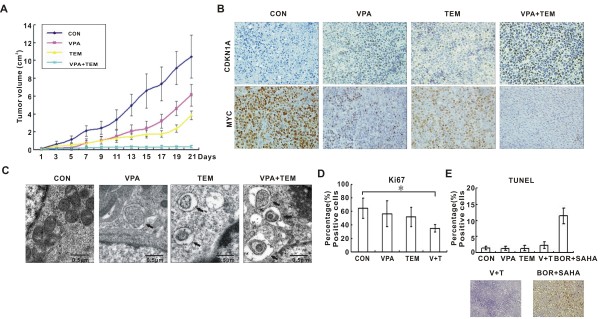
**Valproic acid synergized with temsirolimus in a murine xenograft Burkitt leukemia/lymphoma (BL) model. (A)** Valproic acid (VPA) combined with temsirolimus (TEM) significantly inhibited BL xenograft tumor growth. **(B)** Immunohistochemical study showed that CDKN1A activation is more obvious in VPA-treated groups, and MYC inhibition is most significant in the combination group. **(C)** Ultrastructural study showed increased numbers of autophagic cells in the combination group. **(D** and **E)** Decreased proliferative index Ki67 was identified, **(D)** while TUNEL staining was negative in the combination group. **(E)** The tissue section of the same murine xenograft model co-treated with bortezomib (BOR) and suberoylanilide hydroxamic acid (SAHA) was referred as a positive control.

The in vivo anti-tumor activity of VPA and temsirolimus on BL cells was further evaluated in a murine xenograft model. Subcutaneous inoculation of Namalwa cells into nude mice resulted in a tumor formation at the site of injection in all mice. The sizes of tumors formed in mice co-treated with VPA and temsirolimus were significantly smaller than those of the control and single-agent group after 21 days of treatment (P<0.0001) (Figure 6A).

As in vitro, upregulation of CDKN1A was present in VPA-treated tumors. Inhibition of MYC was more significantly in the combination group than in the single-agent and the control group (Figure 6B). To search for more evidence of tumor cell autophagy, ultrastructure study was performed on mice tumor sections. Compared with those treated with each agent alone, tumor cells in the combination group exhibited increased number of autophagosomes (Figure 6C). Reduced proliferation status of tumor cells was shown by Ki-67 staining (Figure 6D), while terminal deoxytransferase-catalyzed DNA-nick-end labeling (TUNEL) assay revealed no sign of apoptosis (Figure 6E).

## Discussion

Combinations of signal transduction inhibitors are being gradually applied in clinical settings and proven more efficiently than single agent alone to target tumor cells and to avoid acquired resistance [18]. To our knowledge, the present study provided evidence for the first time that the HDAC inhibitor VPA and the MTOR inhibitor temsirolimus, both at a clinically achievable concentration [19,20], interacted synergistically to inhibit BL cell growth. This was found not only in well-established BL cell lines and fresh patient samples, but also in nude mice xenografted with BL cells. Although recent study indicated that VPA can reduce the maximum tolerated dose of temsirolimus in pediatric patients with solid tumors [21], combined treatment appeared to be well tolerated in our study which temsirolimus was administered at a relatively low dose. Of note, the combination exerted the inhibitory effect with a minimal degree of toxicity against normal CD34+ hematopoietic precursors, further confirming their effective and safe role in treating BL.

The observed synergy in cytotoxity, accomplished by combined treatment, mainly resulted from the convergent effect on BL cell autophagy. This was manifested by the ultrastructure study and the autophagy flux assay, and further confirmed by the extent of autophagy being reduced by the pharmacological and molecular autophagic inhibitor. In BL, resistance to chemotherapy is attributed to the inability of tumor cells to die by apoptosis. It may be present at the onset of therapy in high-risk patients, or emerge over time during chemotherapy in relapsed/refractory cases, even after a dramatic initial response. Drugs that target autophagy are efficient in treating BL cells resistant to apoptosis [22,23]. Temsirolimus can induce autophagy in lymphoma cells [24]. Recent reports demonstrated that autophagy appears to be an important therapeutic target of the HDAC inhibitor other than apoptosis in highly proliferative tumors [25,26], which could explain why VPA specifically improve the tumoricidal activity of temsirolimus through promoting autophagy in BL.

Aberrant expression of HDAC1 appears common in tumors, and is associated with enhanced proliferation and defect in autophagy. In liver cancer, targeted disruption of HDAC1 leads to strong anti-proliferative effect and induces autophagic cell death [27]. Our study showed that VPA arrested the G1/S cell cycle transition and activated autophagy through targeting HDAC1, indicating an important underlying mechanism responsible for VPA to interact with temsirolimus to positively regulate BL cell autophagy.

Resistance to MTOR inhibitors is due to feedback AKT activation [11]. The HDAC inhibitor overcomes MTOR inhibitor rapamycin resistance by inhibiting AKT via HDAC3 and potentiates autophagy through downregulation of MTOR pathway [28,29]. In our study, VPA reduced HDAC3 activity and subsequently inhibited AKT phosphorylation induced by temsirolimus. In addition to temsirolimus that directly hits MTOR, VPA modulates the upstream HDAC3 and inhibits MTOR in a rapamycin-independent manner [30]. Aiming for the same pathway with molecules targeting different sites of the protein, the VPA-temsirolimus combination amplified the blockade of MTOR signaling, resulting in further induction of autophagy in BL.

The BL oncoprotein MYC is the key regulatory element by MTOR pathway [31]. Furthermore, MYC mitigates response to the MTOR inhibitor through 4EBP1-mediated inhibition of autophagy [32]. VPA combined with temsirolimus potently targeted MYC oncoprotein, suggesting another important therapeutic mechanism of co-treatment in BL. Importantly, MYC-driven DLBCL have recently been identified as a subtype with inferior survival [33]. VPA-temsirolimus combination induced cell autophagy in MYC-expressing DLBCL DB cells as in BL cells, further indicating its therapeutic role on MYC oncoprotein.

## Conclusions

Our findings highlight the value of combining the HDAC inhibitor with the MTOR inhibitor in treating BL. Targeting cell autophagy warrants further investigation as a promising therapeutic strategy for MYC-associated lymphoid malignancies.

## Methods

### Cells and reagents

BL cell lines Namalwa, Raji, Daudi, Ramos and DLBCL cell line DB were available from American Type Culture Collection. Cells were maintained in RPMI-1640 medium, supplemented with 10% heat-inactivated fetal bovine serum in a humidified atmosphere of 95% air and 5% CO_2_ at 37°C. VPA (V3640) and temsirolimus (PZ0020) were from Sigma-Aldrich. 3-Methyladenine (189490), and ZVAD-FMK (219007) were from Merck & Co. Inc. Bafilomycin A1 (sc-201550) was from Santa Cruz Biotechnology.

Fresh BL cells were extracted from the lymph node and bone marrow of patients. CD34+ cells were isolated from human cord blood using CD34 Progenitor Cell Isolation Kit (Miltenyi Biotec Inc.). The study was approved by the Institutional Review Board and informed consent was obtained in accordance with the Declaration of Helsinki.

### MTT reduction assay

To assess growth inhibition, cells were treated with VPA, temsirolimus, either alone or in combination, in a 96-well plate. After 48 h, 0.1 mg MTT (Sigma-Aldrich, M2003) was added to each well. The samples were incubated at 37°C for 4 hours and the absorbance was measured at 490 nm by spectrophotometry.

### Flow cytometric assay

To assess the distribution of nuclear DNA content, cells were collected, washed in PBS and fixed overnight in 75% ethanol at -20°C, treated with 1% RNaseA (Merck, 70856–3) for at least 15 minutes at 37°C and stained with 50 μg/ml propidium iodide. Cell apoptosis was analyzed using ApoAlert ANX-V-FITC Apoptosis Kit (Clontech Laboratories, Inc., 630110). Cell autophagy was analyzed using rabbit anti-human LC3 (Cell signaling, CST4108) as the primary antibody and DyLight 405 labeled anti-rabbit antibody (KPL, KPL072-08-15-06) as the secondary antibody. The flow cytometry data were collected by a FACSCalibur machine (Becton Dickinson) and analyzed by FlowJo software.

### Isobolographic analysis

Determination of the synergistic effect of VPA-temsirolimus combination was performed using the isobologram of Steel-Peckham [34]. Based on dose–response curves of the two agents, three isoeffect curves were constructed. The area surrounded by the isoeffect curves was referred as the envelope of additivity. When the data points fell to the left of the envelope, that is, the combined effect was caused by lower doses of the two agents than was predicted, the combination was regarded as having a synergistic effect. The synergistic effect was further confirmed by the combination index (CI) method described by Chou and Talalay (CalcuSyn software, Biosoft). When at least 80% of CI values for a combination were less than one, the drug combination was considered to be synergistic.

### Small-interfering RNA (siRNA) transfection

Namalwa cells were transfected with ATG5, HDAC1, HDAC3 siGENOME SMARTpool or Non-Targeting pool as a negative control using DharmaFECT2 transfection reagent (Dharmacon) following the manufacturer’s instruction.

### Western blot

Cells were lysed in 200 μl lysis buffer (0.5M Tris–HCl, pH 6.8, 2 mM EDTA, 10% glycerol, 2% SDS and 5% β-mercaptoethanol). Protein extracts (20 μg) were electrophoresed on 10% SDS polyacrylamide gels and transferred to nitrocellulose membranes. Membranes were blocked with 5% non-fat dried milk in Tris buffered saline and incubated for 2 hours at room temperature with appropriate primary antibody, followed by horseradish peroxidase-conjugated secondary antibody. The immunocomplexes were visualized using chemiluminescence phototope-horseradish peroxidase kit. Antibodies against LC3-I/II (4108), phosphorylated MTOR (p-MTOR) (2971), MTOR (2972), phosphorylated 4E binding protein-1 (p-4EBP1) (9456), 4EBP1 (9644), phosphorylated P70 ribosomal S6 kinase (p-P70S6K) (9205), P70S6K (2708), HDAC3 (3949), HDAC4 (5392), phosphorylated AKT (p-AKT) (4060), AKT (9272), ACTB (4970), c-caspase-3 (9664), c-PARP (9541) and chemiluminescence phototope-horseradish peroxidase kit (7003) were obtained from Cell Signaling. Antibodies against BECN1 (ab51031), MYC (ab28842), HDAC1 (ab150399) and HDAC2 (ab32117) were from Abcam. Anti-P62 antibody (BML-PW9860) was from Enzo Life Sciences, Inc. Horseradish peroxidase-conjugated goat anti-mouse-IgG (sc-2005) and goat anti-rabbit-IgG (sc-2004) antibodies were from Santa Cruz Biotechnology. ACTB was used to ensure equivalent protein loading.

### Enzyme-linked immunosorbent assay

Enzymatic activity of HDAC1 and HDAC3 in lymphoma cells were quantified by enzyme-linked immunosorbent assay using nonisotopic HDAC (BioVision, K331-100) colorimetric kits according to manufacturer’s instructions.

### Transmission electron microscopy

Cells and tissue samples were fixed overnight in 2% glutaraldehyde at 4°C, washed in 0.1M cacodylate buffer, postfixed in 1% osmium tetroxide for 1 hour at 4°C, dehydrated in graded ethanol and embedded in Epon 812 (TAAB Laboratories). Ultrathin sections were prepared, collected on copper grids, stained with uranyl acetate and lead citrate, and examined on electron microscopy (Philips CM120). Ultrastructural studies were focused on double membrane-bound autophagic vesicles named autophagosomes, a gold standard for autophagy.

### Immunohistochemistry and immunofluorescence

Immunohistochemistry was performed on 5μm-paraffin sections with an indirect immunoperoxidase method using antibodies against CDKN1A and MYC. Immunofluorescence was performed on methanol-fixed cells using anti-BECN1 and anti-P62 as primary antibodies, and diaminotriazinylaminofluorescein-labeled donkey anti-rabbit-IgG antibodies (Abcam, ab6800) as the second antibody.

### Murine model

Nude mice (5-6-week-old) were obtained from Shanghai Laboratory Animal Center and injected subcutaneously with 7×10^6^ Namalwa cells into the right flank. Treatments (10 mice per group) were started after tumor became about 0.5 cm × 0.5 cm in surface (day 0). The control group received dimethyl sulfoxide, while the other three groups received for 21 days oral VPA (0.4%w/v in the drinking water daily), intraperitoneal temsirolimus (5 mg/kg every other day), or in combination, respectively. Tumor volumes were calculated as 0.5 × a × b^2^, where ‘a’ is the length and ‘b’ is the width.

### Terminal deoxytransferase-catalyzed DNA-nick-end labeling (TUNEL) assay

In situ cell apoptosis was confirmed by detection of fragmented DNA, using TUNEL assay, on 5 μm-paraffin sections, using DeadEnd Colorimetric TUNEL System (Promega Corporation, G7360) according to the manufacturer’s instruction. The tissue section of the same murine xenograft model co-treated with bortezomib and SAHA was referred as a positive control, as previously described by our study [34].

### Statistical analysis

All assays were set up in triplicate and the results were expressed as the mean±S.D. of data obtained from three separate experiments. T-test was applied to compare two normally distributed groups and Bonferroni to perform multiple comparison. P<0.05 was considered statistically significant. All statistical analyses were evaluated using Statistical Package for the Social Sciences (SPSS) 13.0 software (SPSS Inc.).

## Competing interests

The authors declare that they have no competing interests.

## Authors’ contributions

LHD, SC and ZZ performed the research, LW, ZXS, SJC and WLZ designed the research study, YS analysed the data, and WLZ wrote the paper. All authors have read and approved the final manuscript.

## Supplementary Material

Additional file 1: Figure S1 The synergistic effect of valproic acid (VPA)-temsirolimus combination in Burkitt leukemia/lymphoma (BL) cells. Using the Caclusyn software, most of the data points were presented with combination index (CI) less than one, indicating that the VPA-temsirolimus combination is synergistic in BL cell lines Namalwa, Raji, Daudi and Ramos. Click here for file

Additional file 2: Figure S2 C-caspase-3 and c-PARP expression in Burkitt leukemia/lymphoma (BL) cells treated with valproic acid (VPA) and/or temsirolimus. VPA, either alone or in combination with temsirolimus, did not induce c-caspase-3 and c-PARP expression in BL cells. Click here for file

Additional file 3: Figure S3 Combined effect of suberoylanilide hydroxamic acid (SAHA) and temsirolimus on histone deacetylases (HDACs) in Burkitt leukemia/lymphoma (BL) cells. SAHA, either alone or in combination with temsirolimus, inhibited HDAC1, HDAC2, HDAC3 and HDAC4 expression, in parallel with increased CDKN1A and CDKN1B expression, but decreased p-AKT expression. Click here for file
