# Supplementary figures and images for: Histone deacetylase inhibitor potentiated the ability of MTOR inhibitor to induce autophagic cell death in Burkitt leukemia/lymphoma
Source: J Hematol Oncol. 2013 Jul 18;6:53. doi: 10.1186/1756-8722-6-53 (PMC3722002; doi:10.1186/1756-8722-6-53)

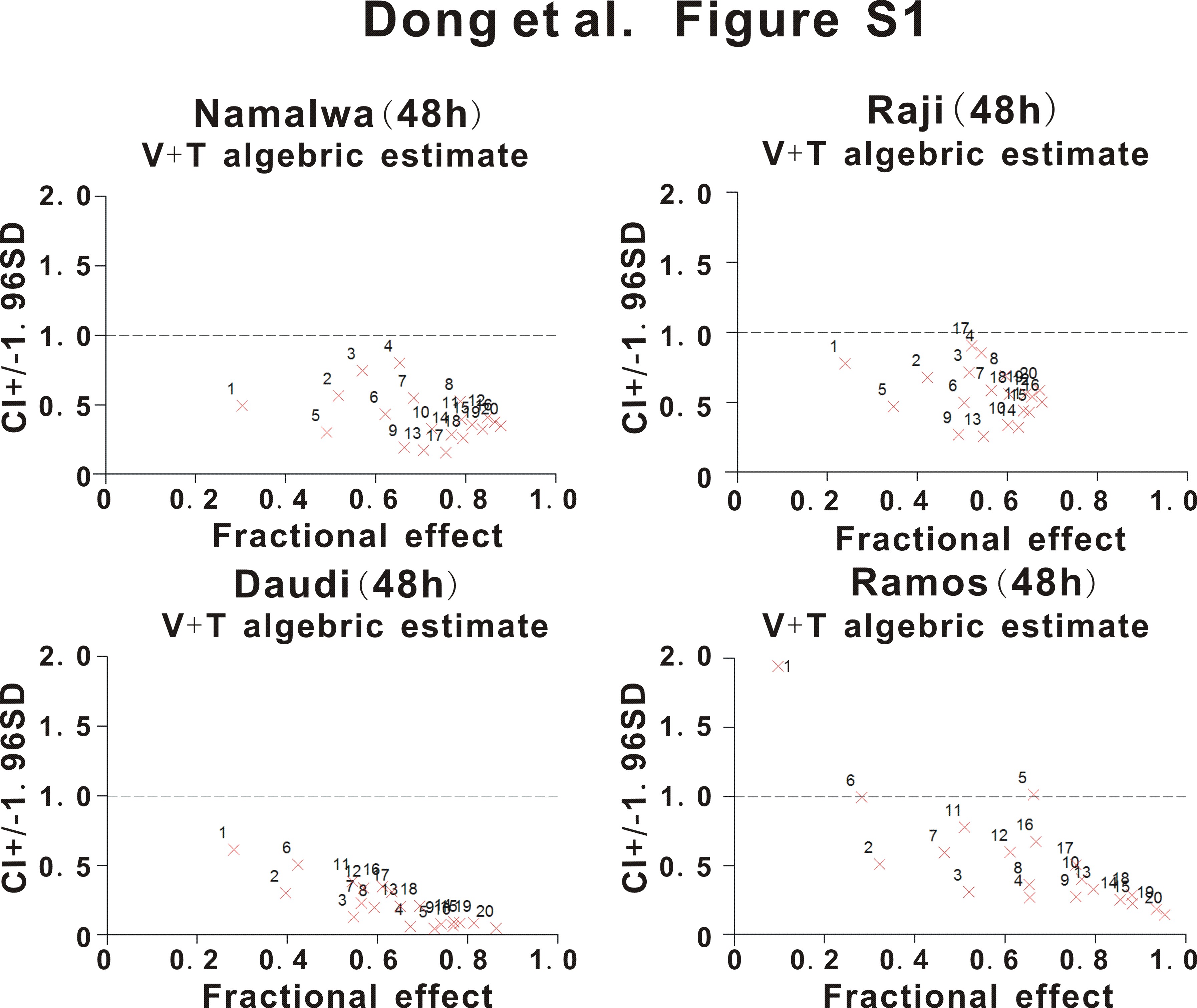

Supplement: Additional file 1: Figure S1 — The synergistic effect of valproic acid (VPA)-temsirolimus combination in Burkitt leukemia/lymphoma (BL) cells. Using the Caclusyn software, most of the data points were presented with combination index (CI) less than one, indicating that the VPA-temsirolimus combination is synergistic in BL cell lines Namalwa, Raji, Daudi and Ramos. [file 1756-8722-6-53-S1.jpeg]

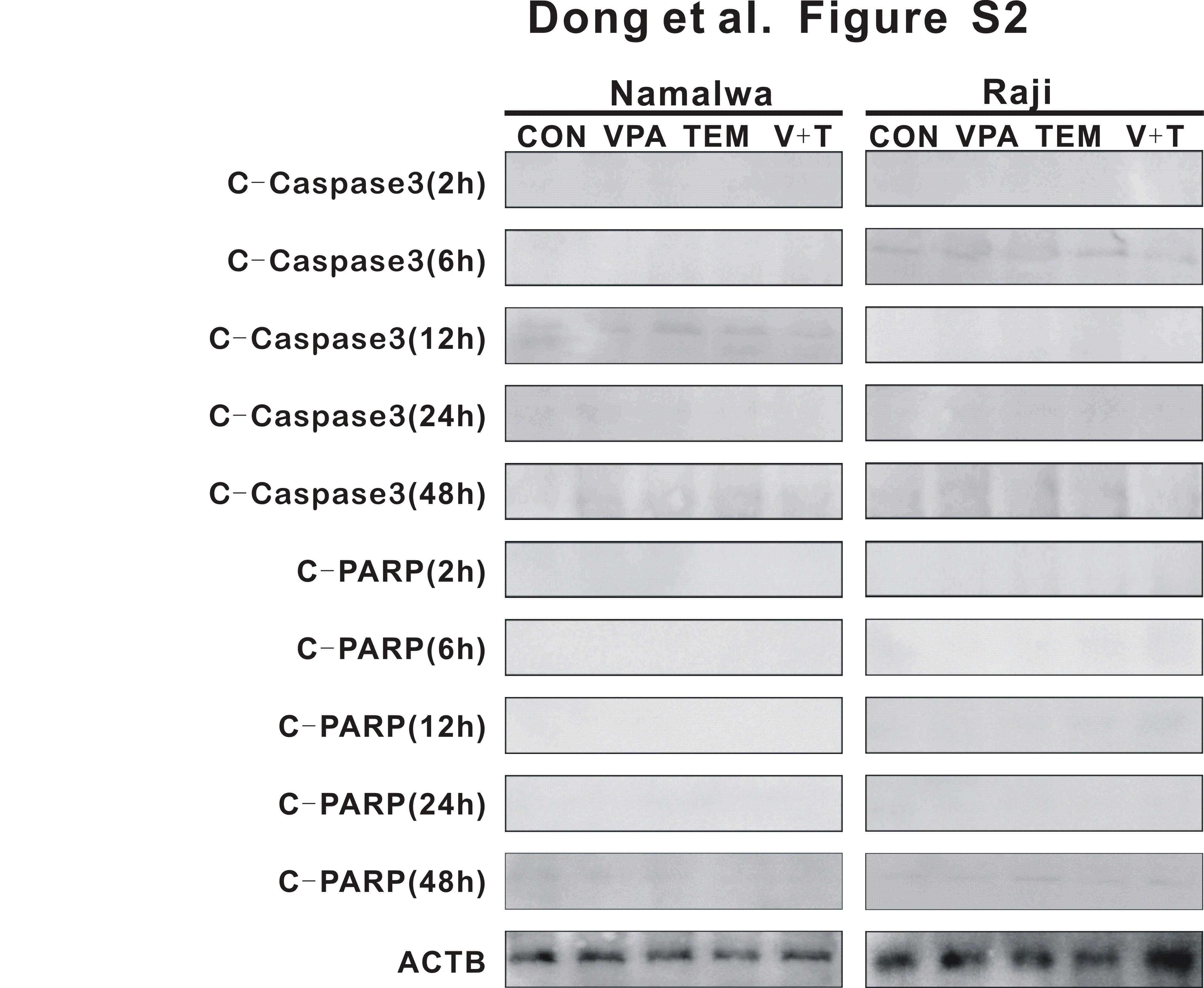

Supplement: Additional file 2: Figure S2 — C-caspase-3 and c-PARP expression in Burkitt leukemia/lymphoma (BL) cells treated with valproic acid (VPA) and/or temsirolimus. VPA, either alone or in combination with temsirolimus, did not induce c-caspase-3 and c-PARP expression in BL cells. [file 1756-8722-6-53-S2.jpeg]

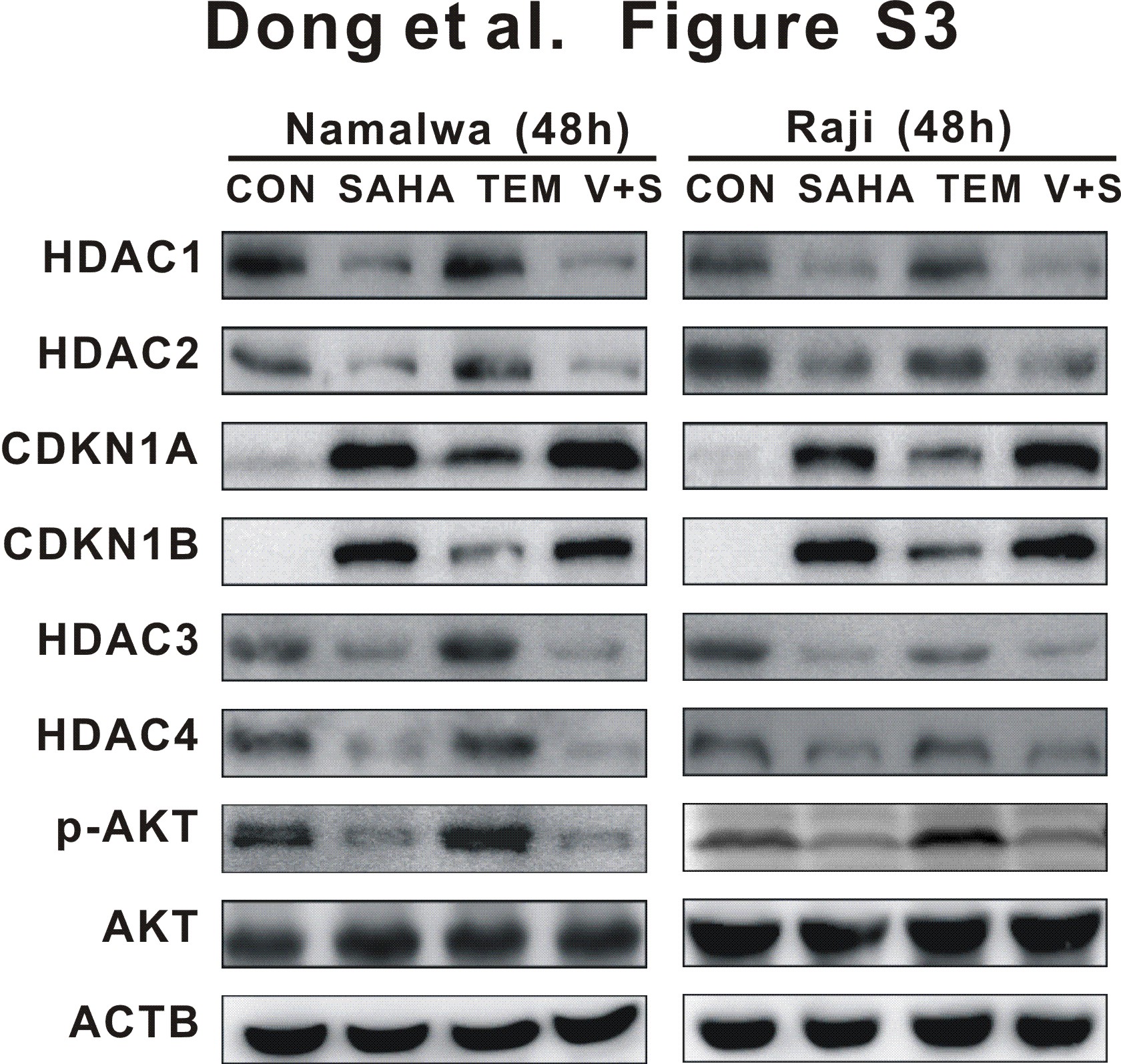

Supplement: Additional file 3: Figure S3 — Combined effect of suberoylanilide hydroxamic acid (SAHA) and temsirolimus on histone deacetylases (HDACs) in Burkitt leukemia/lymphoma (BL) cells. SAHA, either alone or in combination with temsirolimus, inhibited HDAC1, HDAC2, HDAC3 and HDAC4 expression, in parallel with increased CDKN1A and CDKN1B expression, but decreased p-AKT expression. [file 1756-8722-6-53-S3.jpeg]
